# Coping with Pokes: Child, Caregiver, and Clinician Feedback on a Caregiver-Led Educational Resource for Managing Children’s Needle Fear

**DOI:** 10.3390/nursrep16010031

**Published:** 2026-01-20

**Authors:** Hiba Nauman, Emma E. Truffyn, Anna Taddio, Kathryn A. Birnie, C. Meghan McMurtry

**Affiliations:** 1 Department of Psychology, University of Guelph, Guelph, ON N1G 2W1, Canada; etruffyn@uoguelph.ca (E.E.T.); cmcmurtr@uoguelph.ca (C.M.M.); 2 Leslie Dan Faculty of Pharmacy, University of Toronto, Toronto, ON M5S 3M2, Canada; 3 Department of Anesthesiology, Perioperative and Pain Medicine, Cumming School of Medicine, University of Calgary, Calgary, AB T2N 1N4, Canada; 4 Department of Pediatrics, Schulich School of Medicine & Dentistry, Western University, London, ON N6A 3K7, Canada; 5 Pediatric Chronic Pain Program, McMaster Children’s Hospital, Hamilton, ON L8N 3Z5, Canada; 6 Department of Anesthesia, McMaster University, Hamilton, ON L8N 3Z5, Canada

**Keywords:** needle fear, pain, children, vaccination, bibliotherapy, nursing

## Abstract

**Background/Objectives:** Given the critical role of vaccinations and venipunctures in disease prevention and health monitoring, it is concerning that over half of children ages 4 to 8 experience some level of needle fear. Higher levels of fear result in longer procedure times, ineffective pain management, distressing memories of needles, and ultimately, healthcare avoidance. Exposure-based therapy with a therapist is recommended for high levels of fear. However, access is limited due to cost, wait times, clinician shortages, system barriers, and social stigma. Thus, there is a need for an evidence-informed, caregiver-directed educational resource for management of moderate to high needle fear in young children. **Methods:** To address this gap, such a resource was drafted which included a caregiver guide and an illustrated children’s book. The current objective was to gather key user feedback on this initial version of the resource. Participants reported their perceptions of the content, coping strategies, design, organization, and accessibility of the resource through semi-structured interviews and limited quantitative ratings. Participants were children with moderate to high levels of needle fear (N = 6), their caregivers (N = 6), and healthcare professionals (N = 6; including needle providers, child life specialists, and mental health clinicians). Interviews were coded with inductive content analysis; descriptive statistics were calculated for quantitative ratings. **Results:** Participants reported satisfaction with the e-resource and highlighted strengths (e.g., CARD^TM^ system, children’s book) and improvement areas (e.g., length, language). **Conclusion:** Feedback informed revisions to the e-resource in preparation for further evaluation in a follow-up study.

## 1. Introduction

Beyond the over two dozen needle procedures children may receive before 18 years for the prevention of infectious diseases, children also need needles for monitoring their health (e.g., blood draws, finger stick tests) or receiving critical treatment (e.g., insulin injections, intravenous cannulations) [1]. Given the frequency and importance of childhood needles, it is concerning that over half of children ages 4 to 8 report being fearful of needles to some extent, with approximately 30% self-reporting high levels of fear [2]. High levels of needle fear can result in a cycle of traumatic needle experiences: increased pain, behaviors (e.g., trying to escape) resulting in longer procedure times and the use of restraint, and distressing memories [1,2,3,4]. High needle fear also leads to avoidance; indeed, needle pain and fear are barriers to vaccination in 5 to 13% of children under 18 years [5].

Without intervention, highly fearful children can develop into adults who not only avoid critical needles such as vaccinations but also other aspects of healthcare, as seen in Blood-Injury Injection (BII) Phobia [6]. Indeed, 5–10% of adults refuse or delay vaccinations due to needle fear [7,8,9], underscoring why childhood fear must urgently be addressed as a form of prevention. Beyond the genetic components of this high fear/phobia, research suggests that these adults may model fear to their own children thus perpetuating a harmful cycle of fear and healthcare avoidance across generations [1]. Pain is positively correlated with fear [1]; yet children are also fearful of multiple factors beyond pain such as the process of the practitioner searching for a vein, the possibility of receiving multiple pokes, viewing the needle go in/out of the arm, and seeing blood during the procedure [10]. Multiple and/or high fears may lead to pain management that is ineffective, prompting a need for the fears to be managed first.

Existing systematic reviews and clinical practice guidelines summarize evidence-based strategies to improve needle procedures for children [11,12,13,14]. These strategies can be organized into the “5P’s”, psychological (e.g., distraction), physical (e.g., sitting upright), procedural (e.g., most painful needle last), pharmacological (e.g., topical anesthetics), and process interventions (e.g., education for children, parents, clinicians), for low to moderate levels of fear and pain [11]. The CARD™ System is one example of a knowledge translation tool derived from clinical practice guidelines and allows children to self-select strategies to use during their needle procedure (i.e., “Comfort”, “Ask”, “Relax”, and “Distract”). CARD^TM^ has demonstrated effectiveness and utility across a variety of settings including schools, pharmacies, and mass vaccination clinics [15,16,17]. For higher levels of needle fear in children aged 7 and above, more intensive psychological intervention known as Exposure Based Therapy (EBT) is recommended [12]. EBT is considered the gold standard treatment for anxiety and specific phobias, including BII [18,19]. EBT is critically needed because the aforementioned 5P’s pain management strategies, while helpful for many children on the needle fear spectrum, may be ineffective for those with higher levels of fear [1,11,14].

As summarized above, although guidelines exist to improve needle procedures across the spectrum of fear, many strategies rely on needle providers to have prior knowledge or training to implement them, require parents to locate evidence-based strategies across multiple resources, or are inaccessible due to cost or time barriers. EBT in particular, which is typically facilitated by a trained mental health provider, is associated with significant cost, lengthy wait lists, and lack of trained professionals [8]. A stepped-care approach, which would involve provision of accessible self-guided resources could help to address needle fear in some children and prevent the need to pursue provider-led treatment [12,20].

One approach that has been used to provide children with information to help cope with needles is bibliotherapy. However, a recent rapid review found that current bibliotherapy options for children with needle fear are lacking in several areas (e.g., absence of procedural information) [21]. This is problematic as children endorsing higher levels of fear desire more preparatory information [22]. Further, only about 1 in 6 books provided Supplementary Information to caregivers [21], which is unfortunate because caregivers are uniquely set up to support their child before, during, and after the procedure. Indeed, interventions involving thorough parent participation may result in greater improvements in child anxiety and use of coping strategies than child-limited interventions [23,24]. While educational videos and pamphlets targeting caregivers have been created [25], caregivers need to look through multiple sources of information and discriminate between evidence-based information and strategies with no research evidence. Bibliotherapy for children combined with a “parent guidebook” incorporating exposure-based strategies has been used to reduce children’s nighttime fears [26]. Yet no such intervention exists to reduce needle fear in young children.

To address this critical gap, an evidence-informed educational resource for the management of needle fear in young children was drafted by healthcare professionals (2 clinical psychologists, 1 pharmacist); these individuals have specific expertise in anxiety, pediatric pain, and needle fear, who were leaders in the aforementioned clinical practice guidelines and systematic reviews. This e-resource consisted of two components: a story book for children and an informational guide for caregivers. Together, they provided strategies from existing clinical practice guidelines [11,14] for (1) low to moderate levels of needle fear (e.g., CARD^TM^, children’s book), and (2) moderate to high levels of needle fear (e.g., principles of EBT). The aim of this study was to gather feedback from caregivers, children, needle providers, child-life specialists, and mental health professionals on each component of the e-resource. Specific objectives involved examining participants’ perceptions of the draft e-resource regarding: (1) content and strategies related to coping with needle pain and fear; (2) the design, organization, and accessibility (i.e., how easy the e-resource is to understand and use); (3) the e-resource overall (satisfaction). No specific hypotheses were made.

## 2. Method

### 2.1. Study Design

This descriptive study involved a predominantly qualitative design within a realist, essentialist approach which assumes that participants’ feedback reflects and describes their genuine thoughts and feelings about the e-resource. Semi-structured interviews were the primary data source. Participants also completed a brief feedback survey that included a small number of author-generated, open-ended questions and quantitative ratings. Open-ended survey responses were reviewed but were not systematically coded or analyzed, as they did not provide new or differing information when compared to the interview data. Quantitative ratings are included when relevant to contextualize participant feedback; however, they were not integrated with qualitative findings, and no triangulation procedures were conducted. This study represents the first step in a series of research studies aimed at developing and evaluating the e-resource. The e-resource was specifically created for this study and had not been previously evaluated or used in research settings.

### 2.2. Participants

To best highlight the needs of children with moderate to high levels of needle fear, participants with expertise in delivering needles, treating needle fear and anxiety in children, and/or lived experience with needle fear were sought. The latter included 5 to 8-year-old children with moderate to high levels of needle fear and their caregivers. The lower limit of 5 years was selected because children ages 4 and under have been found to have limited ability to report on future internal states that are different from their current state [27]. Additionally, a purposive sample of 3 types of healthcare professionals were recruited: needle providers (e.g., nurses, pharmacists), mental health professionals (e.g., clinical psychologists), and child-life specialists. An a priori sample size of 12–18 interviews was planned based on the “information power” of our sample. That is, our study had a narrow aim, participant groups with specific expertise, and anticipated rich data from the detailed interviews, resulting in a small sample to be sufficient [28]. Recruitment occurred via social media, listservs, and outreach to relevant healthcare professionals and organizations. Interested participants completed a Qualtrics survey for eligibility criteria screening (Supplementary Material S1); namely, participants were required to: (1) be Canadian or USA residents; (2) be ≥18 years old (caregivers, healthcare professionals); (3) be proficient in English; (4) have access to technology for e-resource review. In addition to being between 5 and 8 years of age, children were required to have moderate to high levels of needle fear, as rated by their caregivers; the latter was operationalized as a rating of ≥5/10 on a numerical rating scale of needle fear and endorsing at least one of significant distress, history of restraint during a needle procedures, and/or delaying of a needle procedure.

Healthcare professionals had to self-report registration with their professional organization to work clinically in their region; mental health professionals had to self-report expertise in treating children with needle fear using EBT. The Qualtrics screening survey also automatically collected participants’ GeoIP location to exclude those outside of Canada or the USA. To verify participant identity and rule out the possibility of fraudulent responders [29], all eligible participants were also required to: (1) have a valid USA/Canadian phone number; (2) participate in a screening phone call to confirm their eligibility; (3) participate in a second phone call to answer knowledge questions related to the e-resource; (4) have their camera on for their virtual interview. Recruitment continued until both the desired sample size and repetition of key ideas/codes was reached.

### 2.3. Ethical Considerations

Research Ethics Board approval was granted by the University of Guelph (REB#22-08-024), Assent was obtained from all child participants; consent was obtained from caregivers and healthcare professionals. Participation was voluntary, and participants (including children) were allowed to leave or end the interview at any time. There were no anticipated risks to participating in this study beyond what would be encountered in the participants’ everyday lives. It is possible that participants may have felt self-conscious in sharing their ideas with the interviewers. Participants were told that all responses to the questions posed were solely opinions and there were no “right” or “wrong” answers. Some children may have found some of the questions or discussions about needle fear upsetting or difficult to talk about. Reviewing the e-resource may have benefited participants by providing information as to how children’s needle fears can be managed or by demonstrating some useful ways to explain these concepts to their children/clients/patients. No adverse events occurred during data collection.

## 3. Measures and Materials

### 3.1. Demographics

The demographics questionnaire collected data on participant age, gender (open field), and ethnicity. Caregivers were asked to report on their own demographics as well as their participating child (see Supplementary Material S2).

### 3.2. Needle Fear E-Resource (Participant Feedback Was Sought on This Initial Version)

Prior to data collection, the e-resource was drafted by the senior author/lead researcher’s supervisor (C.M.M.—psychologist) and collaborators (A.T.—pharmacist and K.A.B.—psychologist). All strategies and education within the caregiver guide stemmed from relevant clinical practice guidelines and systematic reviews for management of needle pain and fears led by the developers [11,12,13,14,30]. The e-resource consisted of two parts: Children’s Book (Supplemental Material A) and the Caregiver Guide (See Supplemental Material B). The 19-page children’s book depicted a narrative where children in an elementary school classroom are taught basic information about vaccinations, venipunctures, and coping strategies to manage their needle fear and pain by a visiting nurse and their teacher. Evidence-based strategies (e.g., distraction, deep breathing, topical anesthetics, exposures) are introduced using language and illustrations appropriate for the target audience of 5 to 8-year-old children. The book also included procedural and sensory information.

The caregiver guide aimed to summarize evidence-based fear and pain management strategies in lay language by directing caregivers to the most relevant content depending on the level of their child’s fear. The main components of the 30-page guide included psychoeducation on needle fear and pain, using the CARD^TM^ System, exposures for high levels of fear (i.e., learning, planning, doing, and maintaining exposures), managing dizziness/fainting, and multiple helpful resources (e.g., exposure examples, videos, tracking template). The caregiver guide was purposefully formatted in a “Choose Your Own Adventure” style which allowed caregivers to quickly navigate to the most appropriate and relevant information for their child. Consequently, caregivers were not provided with specific guidelines or instructions on how to use the e-resource as it was designed to allow flexibility rather than require a full read through page by page.

The present study served as the first step in developing the e-resource. The e-resource was created specifically for this study, which is part of a larger series of studies evaluating its effectiveness. Because the e-resource did not previously exist, it had also never been used in research settings prior to this work.

### 3.3. Interview Guides

Interviews were semi-structured and asked participants open-ended questions about the e-resource including what parts were most/least helpful, what they would change, and challenges/barriers (Supplementary Material S3—interview guide). Interview questions were author-generated. Children were asked about their impressions of the children’s book, with parents supplementing their answers when necessary.

### 3.4. Feedback Survey

Using 5-point Likert scales (1 = Strongly Disagree; 2 = Disagree; 3 = Neutral; 4 = Agree; 5 = Strongly Agree), the e-resource feedback survey (Supplementary Material S4) gathered ratings of satisfaction with the e-resource, understanding of the information presented, and accessibility of the resource. Participants were also asked to estimate how long they engaged with the e-resource. The surveys also included a small number of author-generated, open-ended questions. Responses to these questions were not systematically coded or analyzed as there was no new or conflicting information when compared to interview responses.

### 3.5. Procedure

Data collection took place from September 2023 to January 2024. After completing the screening survey and written consent (assent for children), eligible participants were called by the lead researcher (H.N.) to verify their identity and screen out fraudulent participants [29]. Each eligible adult participant then completed an online demographic survey and were provided access to the e-resource on a password-protected website. Following review of the e-resource, H.N. called participants to complete a brief knowledge check to ensure they had reviewed the full materials. An interview, hosted virtually on Microsoft Teams (v2024) for convenience and data security was then scheduled. After affirming verbal consent/assent from all participants, including children, detailed feedback was gathered by H.N. and a co-author (E.E.T.) who are PhD candidates in clinical child psychology, with training in clinical interviewing. Interviews were between 45 min to an hour in duration. Depending on child comfort, they were interviewed with their caregiver present either before or after the caregiver-focused questions. Adult participants then completed the brief feedback survey.

### 3.6. Analyses: Qualitative Coding, Trustworthiness, and Descriptive Statistics

Interviews were transcribed verbatim by trained research assistants using audio-video recordings and automatically generated transcripts with all identifying information removed. The transcripts were then reviewed for accuracy by research assistants and the lead researcher (H.N.). NVivo SoftwareTM v.14 was used to analyze the interviews using inductive content analysis [31]. The unit of analysis was a given interview, and only manifest content was analyzed. The coding process included three phases: preparation, organization, and reporting [31]. To enhance trustworthiness, H.N. completed Elo and colleagues’ [31] checklist which involved H.N. reading through each interview carefully during the preparation phase to get a general sense of the data. H.N. then immersed herself in the data and thoroughly reviewed transcripts multiple times. In the organization phase, H.N. engaged in open coding and made detailed notes and headings before generating categories. H.N. consulted with C.M.M. and E.E.T. to refine the categories and reorganize according to similarities and differences, resulting in the final categories presented in the results. The COREQ checklist was also utilized to improve the transparency of the findings [32]. Descriptive statistics within IBM SPSS (v 29.0.2) were used to analyze quantitative data from the feedback survey (i.e., frequencies, central tendency, and variability).

## 4. Results

### 4.1. Participants

A total of 18 participants completed the study. Demographic information can be found in Table 1. One child refused to participate in the interview portion of the study. Their caregiver was able to describe their experience reading the children’s book, and their demographics are included in Table 1. Additionally, 1 caregiver and 3 healthcare professionals enrolled but did not complete the study for unknown reasons.

### 4.2. Results

Results are presented within the following broad categories: content and strategies, design and organization, accessibility, and satisfaction. Sample quotes illustrating the findings are displayed in tables. Unless otherwise noted, a result pertains to the entire resource (vs. solely the children’s book or the caregiver guide). Participant groups will be referred to as: Caregiver (Cg); Child (Ch); Needle Provider (NP); Mental Health Professional (MHP); Child Life Specialist (CLS). While examining differences in perceptions across participant groups was not an aim, notable differences are highlighted when relevant. Children provided feedback on only the children’s book whereas all adult participants provided feedback on the entire e-resource.

#### 4.2.1. Content & Strategies

Participant feedback pertaining to the educational content within the e-resource can be categorized into needle pain and low to moderate fear management strategies (herein “pain management strategies”; e.g., CARD^TM^ system, topical anesthetics) and moderate to high fear management strategies (herein “high fear management strategies”; e.g., exposure) (Table 2). The majority of participants, including children, highlighted CARD^TM^ and the strategies within it as helpful. Many participants also highlighted that conceptually learning about exposures or “facing fears” was helpful. Participants from healthcare professional groups (N = 6) indicated in the feedback survey that they believed the information presented was accurate in both the caregiver guide (*M* = 4.3, *Mdn* = 4.5, SD = 0.82, IQR = 1.35 Range = 3–5) and the children’s book (*M* = 4.5, *Mdn* = 4.5, SD = 0.56, IQR = 1.00, Range = 4–5).

#### 4.2.2. Design & Organization

Caregiver and healthcare professional groups (N = 12) provided feedback on the visuals of both components of the e-resource (e.g., illustrations, diagrams, layout) and their ability to navigate it (e.g., organization, links) (Table 2). Ratings reflected strong organization of information in both the caregiver guide (*M* = 4.4, *Mdn* = 5.0, SD = 0.83, IQR = 1.75, Range = 3–5) and children’s book (*M* = 4.2, *Mdn* = 4.0, SD = 0.79, IQR= 1.00, Range = 3–5). Regarding children’s book illustrations, participants reported mixed feelings on the cartoon needle, with some children and adults reporting it was too scary, and other caregivers reporting their child was not fearful of it.

#### 4.2.3. Accessibility

Caregiver and healthcare professional participant groups expressed mixed views about how easy the e-resource is to understand and use, what demographic the e-resource is a good fit for (e.g., child age, parent education level), and what barriers to accessibility may be present (Table 3). According to the feedback survey, caregivers and healthcare professionals (N = 12) believed that the caregiver guide (*M* = 4.6, *Mdn* = 5.0, SD = 0.67, IQR = 1.00, Range = 3–5) and children’s book (*M* = 4.3, *Mdn* = 4.5, SD = 0.87, IQR = 1.75, Range = 3–5) had a good amount of detail. More than half of these participants (58.3%) also found that the amount of information within the caregiver guide was “too much”, whereas the remaining 41.7% found it to be the “right amount”. In contrast, 25% participants found the children’s book to be “too much”, 66.7% found there to be the “right amount” of information, and 8.3% found it to be “not enough”. Caregiver and healthcare professional groups (N = 12) reported that the information in the caregiver guide (*M* = 3.6, *Mdn* = 4.0, SD = 1.16, IQR = 1.00 Range = 1–5) and children’s book (*M* = 4.1, *Mdn* = 4.0, SD = 1.00, IQR = 1.75, Range = 2–5) was clear and understandable. On average, caregivers and healthcare professionals spent approximately 68 min reviewing the e-resource as a whole.

Caregivers and healthcare professionals generally had mixed opinions on the ideal age range for the children’s book. On the high end of the age range, the caregiver of an 8-year-old reported that their child was seeking more advanced information than the children’s book could offer (e.g., scientific information about vaccinations). Participants also expressed curiosity around how a caregiver would know about the e-resource and how to find it online which raised questions about how the e-resource should be disseminated.

#### 4.2.4. Use & Satisfaction

Participants reported on their satisfaction with the e-resource as well as their emotional experiences while reviewing the materials (Table 4). Multiple participants reported on how the caregiver guide “normalized” the prevalence of higher levels of needle fear. However, many children shared brief expressions of fear (e.g., “too scary”), or pain (e.g., “owie”) when asked to reflect on their experience reading the children’s story. Participants described a number of uses and applications of the e-resource as a whole or in parts. They also reported on how likely they were to recommend the caregiver guide and children’s book to others (Table 5).

Caregiver and healthcare professional groups shared their satisfaction with the organization of the e-resource and appreciated that it could be used in different ways (e.g., to reinforce what they know, to learn new strategies, etc.). Participants also shared positive comments about the children’s book storyline and illustrations. The feedback survey results reflected that most caregivers and healthcare professionals were likely to recommend the e-resource to other parents and healthcare professionals. However, a few participants reported being “unsure” about recommending the e-resource and the likelihood of caregivers following its recommendations due to length and potential to “overwhelm”. Despite these concerns, more than half of the participants reported being “very satisfied” with the children’s book and “moderately satisfied” with the caregiver guide (Table 5). Further, caregivers (N = 6) agreed that the information in both the caregiver resource (*M* = 4.5, *Mdn* = 5.0, SD = 0.83, IQR = 1.25, Range = 4–5) and children’s book (*M* = 4.0, *Mdn* = 4.5, SD = 1.26, IQR= 2.25, Range = 2–5) were applicable to them and their child.

## 5. Discussion

The present study is part of a line of research to develop and evaluate a free, low intensity, and caregiver-directed resource for managing moderate to high needle fear in children. The present study aimed to gather feedback from children, caregivers, and healthcare professionals to improve the e-resource prior to a follow-up study evaluating the e-resource’s effectiveness. Overall, participant qualitative and quantitative feedback highlighted several strengths of the e-resource. Regarding the children’s book, participants appreciated the narrative and reflected positively on the inclusivity of the characters. However, children themselves consistently shared expressions of fear or pain when reflecting broadly on their experience reading the story. While this may imply that children within our sample did not like the illustrations or narrative, their reaction is also understandable given our purposive sample of moderate to highly fearful children. It is likely that the children’s book served as an “exposure” for our sample. Within the caregiver guide, participants found CARD^TM^ to be particularly helpful and easy to utilize. This is consistent with the literature which also highlights the utility of CARD^TM^ and its function in supporting children during needle procedures [15,16,17]. Another notable strength was the validation caregivers felt from the e-resource’s non-pathologizing language and from learning about the prevalence of high levels of needle fear. Participants positively rated the e-resource’s organization, accuracy, applicability, and accessibility, and expressed high levels of satisfaction overall.

When reflecting broadly on the caregiver guide, most participants found the caregiver guide to be too lengthy and overwhelming for caregivers of 5–8-year-old children. Given that unsuitable length and lack of time are barriers to engaging with self-guided digital interventions [33], results highlighted the need to shorten the caregiver guide. However, the majority of participants had difficulty identifying content that was “unhelpful” or should be removed. This contradictory finding emphasized the need to restructure the e-resource in a manner that retained key information but was briefer and more engaging. Existing literature also recommends digestible content and design to support user engagement in self-guided interventions [33]. A key aspect of the caregiver guide was also content on exposures or “facing fears”. Participants expressed mixed feelings with some highlighting it as helpful, but others concerned it may be “overwhelming” for caregivers without higher levels of education. While additional research is needed to examine how content may be perceived by caregivers with lower levels of literacy, parent-guided exposure-based interventions in extant literature have demonstrated some utility [26]. As the e-resource is primarily caregiver-directed and lacking the external accountability of traditional therapy, it may require caregivers to be highly self-motivated, particularly for intensive content such as exposures [33,34]. Despite this, research suggests caregivers are interested in evidence-based tools specifically geared towards them as they often struggle to identify and access high quality information [35].

Based on the aforementioned participant feedback, a number of revisions were made to the e-resource. Caregiver guide revisions involved breaking up text into shorter bullet points, simplifying language, and linking to videos/interactive handouts when possible. Content was made more digestible by ending modules with bullet point summaries of key information. Anticipating that caregivers of young children may be busy or overwhelmed, a section was added normalizing adult needle fear, caregiver anxiety around needle procedures, and also providing self-care strategies [7,8]. Revisions to the children’s book involved making the content more developmentally appropriate by simplifying language, reducing the amount of content per page, and adding “bright and friendly” colors. Slight changes were made to the illustrations to make the characters appear more “cartoon-like” and character expressions were connected to feelings mentioned in the story (e.g., worried facial expression when asking about needles). The final e-resource can be found on the Pediatric Pain, Health, and Communication Lab website (https://pphc.uoguelph.ca/resources/child-needle-fear-e-resource/).

## 6. Strengths, Limitations, and Future Directions

To our knowledge, this is the first study to: (a) develop a resource for needle fear and pain that aims to equip both children and caregivers with evidence-based information and coping strategies using bibliotherapy and thorough caregiver education; (b) target children with moderate to high levels of fear with a low intensity resource that is free, accessible online, and led by caregivers at home rather than mental health professionals in a clinic; (c) seek feedback from all relevant groups (children, caregivers, needle providers, child life specialists, mental health professionals) in the development of the e-resource. The valuable feedback gathered through this study guided detailed revisions of the e-resource, which will undergo further evaluation. Ultimately, the future goals of the resource will be to reduce children’s needle fear and pain, bolster caregivers’ knowledge and confidence, train healthcare professionals on evidence-based strategies, and serve as a resource in therapy to supplement treatment.

Despite these strengths, there are a number of limitations to consider. Firstly, the data represent attitudes and opinions of the participants recruited. Despite consistencies within and between groups, it is possible that other caregivers, children, and healthcare professionals may hold different perspectives on the e-resource material. Secondly, given the nature of several of our key groups (i.e., NPs, MHPs, CLSs), our sample consisted of many participants with higher education and prior exposure to concepts within the e-resource. Caregiver education level was also not collected. As such, our sample may not have been well positioned to judge the accessibility of the language for families with lower literacy. Future studies should aim to explore the e-resource’s accessibility across families with different levels of education. Additionally, participants were recruited from the United States and Canada; although the health systems differ between these countries, findings may have limited the applicability of findings to regions with other healthcare systems and cultural contexts. Future research should examine the applicability of the e-resource in more diverse and international settings. Further, as developmental abilities of children ages 5 to 8 can vary, it was difficult to capture the suitability of the children’s book for our age range. Anecdotally, the interviewers noticed that children on the higher end of the age range could more effectively engage in a discussion of what they learned from the book. Based on these observations, we extended our age range to 5- to 9-year-old children for the follow up evaluation of the e-resource. Additional research is still needed to fully grasp children’s ability to learn coping strategies via the e-resource and then successfully applying them during needle appointments.

## 7. Conclusions and Implications

In summary, this study serves as a first step in the development of a needle fear e-resource that targets children with moderate to high fear and their caregivers. The creation of this e-resource is consistent with the stepped care approach, a model supporting the use of services at lower levels of treatment intensity and “stepping up” to higher levels as needed. The e-resource aims to do so by providing caregivers with a free and accessible alternative to therapy and by addressing a critical treatment gap for children with higher levels of needle fear. Although preliminary, the positive feedback from participants suggests that the resource may be promising for supporting children and caregivers during needle procedures. Constructive feedback provided by participants resulted in significant revisions to the final draft of the e-resource which has been further evaluated by children and caregivers in a follow-up study that utilized a pre-post study design. Ultimately, the current study lays the foundation for continued evaluation of the e-resource and provides researchers with an understanding of what fearful children, caregivers, and healthcare professionals believe would best help children who struggle with needles. This can inspire further interventions targeting other groups (e.g., training interventions for healthcare providers, interventions for older children undergoing school-based immunizations).

## Figures and Tables

**Table 1:** Participant Demographic Information.

Participant Group	N	Gender	Age (M_age_, Range)	Ethnicity
Caregivers	6 (100% mothers)	100% female	39.0 (32–44)	83.3% White/European 16.7% Southeast Asian
Children	6	66.7% male	6.3 (5–8)	50% White/European, 33.3% Mixed Asian 16.7% First Nations/Métis/Inuit
Healthcare Professionals	6 Total 2 needle providers (nurses) 2 mental health professionals (1 clinical psychologist, 1 psychotherapist) 2 child life specialists	100% female	42.3 (37–54)	83.3% White/European 16.7% Métis/Inuit/First Nations

**Table 2:** Participant Perceptions of E-Resource Content, Strategies, Design, & Organization.

Category	Sample Quotes
Pain Management Strategies	*“And you liked the idea of what, during the needle?” (Cg5)* *“Of watching, like, um playing the video game while I get it, or like watching a show.” (Ch5; age 6)* *“Well, like the examples that you have like are good too—[be]cause some people might not know how to get comfortable so you have all those options of how they can be comfortable and like the questions to ask, like those are, I think, are important to have in here too, right?” (Cg4)*
High Fear Management Strategies	*“And I actually made a thing—like a thing with stairs, it’s called a ladder, but I made it with stairs, and I labelled each stair, like 1 would be putting in the needle—I mean 0 would be putting a needle into a chair, cause I’ve tried that already, and then it didn’t work before, and um number 16, otherwise known as the—the floor above the stairs would be like, getting the needle.” (Ch2; age 8)* *“And then some of the stuff about exposure … I like the diagram of how exposure works, the fear hill, I like that, I think that’s a good visual, but I think there’s probably too much description of exposure overall.” (CLS1)*
Format, Navigation, & Visuals	*“I think working in more—yeah bullets and like bulleted lists or numbered lists, I think. You have—there’s some but there’s also a few sections… like there’s this section about investigate thinking traps by using detective thinking, ask your child questions like, and it’s sort of formatted where there’s like two columns but something like that might read cleaner and easier in just one bulleted list.” (CLS1)* *“I liked the diagrams. I liked that it’s not a big, huge bulk and you’re reading it and just it’s chapter after chapter and page after page of—you know you could almost look at a page and see what it’s giving you.” (Cg2)*
Children’s Book Illustrations	*“I mean right off the bat if—my first initial thought would be as a reactive thought, get rid of that really sharp point that’s on [the needles], um because then those instruments don’t look nearly as threatening, but honestly, I—I’m not really sure.” (MHP2)* *“I liked that they were inclusive. There were pictures of different—I’m assuming different religions and different people with different skin colors perhaps, and people who are differently abled—which I like to see that. I like to see those groups represented.” (Cg5)*

**Table 3:** Participant Perceptions of Accessibility.

Category	Sample Quotes
Language & Terminology	*“It’s just really high level. It’s probably at a healthcare provider, someone with really strong health literacy level—Um I don’t think it’s for a—Not to say most—I mean, some parents are very highly educated, but um, I mean it depends on who you’re trying to target, right?” (Cg1; referring to caregiver guide)* *“The language is good. It is you know, simple words, there’s no complicated words that you know a child wouldn’t be able to sound out. There’s a lot of like repetitive words like “parents”, “doctor”, we use like the same terms, so you know once they realize that says doctor they could keep going throughout.” (Cg2; referring to children’s book)*
Length	*“I found it very overwhelming, because it’s 30 pages long, and I’m like okay, from a perspective of a parent, if my child has anxiety, to be given something like this, I don’t know if I would read it.” (MHP2)* *“So, if there was something like, a summarized, condensed version of it, umm, and then if parents want to know more, right? Like we’ve got the more in-depth information, um, is something that I would maybe see beneficial. Uh, otherwise, I could see parents starting to go through it and being like, oh, I don’t have time for this, it’s too long, and just setting it aside.” (NP1)*
Children’s Book Target Age Range	*“My six-year-old, she could follow along; she enjoyed it. I think because the scenario the book is built around is like sort of being in class and having that discussion, I think it’s going to appeal to school age kids.” (CLS1)* *“Like I think for her age [5], this book was too advanced for her. There’s like too many words. So like when I read it to her, she was like, at the end, she’s like, “I don’t like needles!” That’s all she had to say.” (Cg4)*
Accessibility—Other	*“Uh, yes, we do have a lot of people that don’t have access to the internet, so where something, a paper hand out, but not a huge paper handout, because often we’re, we’re seeing, like our immigrants or very isolated population that may be lower income, so lower education as well, in order to target that population, it would be nice to have something like a paper copy…” (NP1)* *“I didn’t find any barriers with using the resources. It was easy to print off, so I print all of it off. I could easily read it online, but I just liked it kind of like the—the piece of paper in my hand when we were reading at nighttime. Um, so no I didn’t find any issue with that.” (Cg2)*

**Table 4:** Participant Satisfaction with and Perceptions about Use.

Category	Sample Quotes
Applications & Use	*“I think I-for me, I love um -Thinking as a clinician in the hospital setting, I like the versatility of a tool um and that it can be utilized again in whole and in part and it can kind of be individualized based on the needs, so I really did kind of liked that part.” (CLS2)* *“There’s a lot of information and it can be used not even just with needles like it was—we were at just at the dentist and a lot of this can be applied to that anxiety and-and the fear we-we have been dealing with a lot of uhm different anxieties with her. So, it-it is definitely helpful with that.” (Cg3)*
Validation	*“And you know that, but in the moment ‘laughs’ you feel like this only happens to me, so it is nice to just see it so you can remind yourself, you know, that while there may not be another screaming child in this room right now, they were not the only ones today.” (Cg2)* *“I really liked that the kids were—the general consensus among the children was that they didn’t like needles. I feel like that really validated [child’s name] fears.” (Cg5)*
Children’s Emotional Experiences	*“What don’t you like about it, [child’s name]?” (Cg1)* *“It’s owie-“ (Ch1; age 5)* *“Yeah. What don’t you like about it?” (Interviewer)* *“The book is owie.” (Ch1)* *“Like we went through the whole thing, but I would say just cause there are so many words, it was kind of above her head and her consistent thing was “I don’t like needles”, “Needles are scary”, “I don’t want a needle”, like it was just like, “Oh, no, now I have to go get a needle or-“, like, no, no.” (Cg4)*
Satisfaction—Other	*“So, it leads—It’s a great segue into compliment I wanted to give. That hand out number one [list of needle-related situations for exposures] was exceptionally organized, and I thought that would be a very useful and practical handout for me to have to sort of, you know, sometimes I am thinking of different ways for a kiddo to cope and for parents to help.” (MHP1)* *“Getting her feedback on okay is it like, are you afraid of this or is this what’s going on or like I think it’s just in all aspects for her, I think this is going to be really beneficial to work through needles and any other fear or pain that she might experience and might have to go through.” (Cg3)*

**Table 5:** Caregiver & Healthcare Professional Attitudes on Recommending the E-Resource & their Satisfaction (N = 12).

Participant Attitudes		Frequency (%)				
		Very Unlikely	Unlikely	Unsure	Likely	Very Likely
How likely are you to recommend this resource to other parents of children with moderate to high levels of needle fear?		0 (0.0)	0 (0.0)	3 (25.0)	4(33.3)	5 (41.7)
How likely do you think your clients/patients would be to follow the recommendations in the e-resource?		0 (0.0)	0 (0.0)	2 (16.7)	8 (66.7)	2 (16.7)
		Very Dissatisfied	Moderately Dissatisfied	Neutral	Moderately Satisfied	Very Satisfied
Please indicate your overall level of satisfaction with the	Children’s Book	0 (0)	0 (0)	0 (0)	4(33.3)	8 (66.7)
	Caregiver Guide	0 (0)	0 (0)	1 (8.3)	7 (58.3)	4 (33.3)

## Data Availability

Data is not available upon request due to research ethics limitations.

## Supplementary Materials

The following supporting information can be downloaded at: https://www.mdpi.com/article/10.3390/nursrep16010031/s1: S1. Qualtrics screening survey with eligibility criteria, S2. Demographics Questionnaire, S3. Interview guide with questions for all participant groups, and S4. Feedback survey.

## Public Involvement Statement

Children with moderate to high levels of needle fear and their caregivers were directly involved in this research through qualitative interviews aimed at gathering their feedback on the e-resource. Their perspectives informed revisions to improve the content and accessibility of the intervention. In addition, healthcare professionals—including needle providers (nurses), mental health professionals, and child life specialists—provided feedback based on their clinical expertise and experience supporting children during needle procedures. This input helped guide revisions to improve the e-resource for children with needle fear and their families.

## Guidelines and Standards Statement

The Consolidated Criteria for Reporting Qualitative Research (COREQ): a 32-item checklist for interviews and focus groups was used to support in drafting this manuscript [32].
