# Supplementary material for: Coping with Pokes: Child, Caregiver, and Clinician Feedback on a Caregiver-Led Educational Resource for Managing Children’s Needle Fear
Source: Nurs Rep. 2026 Jan 20;16(1):31. doi: 10.3390/nursrep16010031 (PMC12844966; doi:10.3390/nursrep16010031)
Supplement: Supplementary file 1 [file nursrep-16-00031-s001.zip › Child NF Manuscript-Supplementary Materials.pdf]

Supplementary Material S1

Healthcare Professional Screening Questionnaire

1. Are you currently living in Canada or the United States?
   - ❑ Yes
   - ❑ No

2. Are you 18 years of age or older?
   - ❑ Yes
   - ❑ No

3. Are you able to speak, write, and read English well enough to answer some written questions and participate in a discussion in English?
   - ❑ Yes
   - ❑ No

4. *For Child Life Specialists:* Does your profession involve supporting children and/or their families during needle procedures?
   For Mental Health Professionals: Are you a mental health professional with expertise in the treatment of children's anxiety and/or fear related to needles?
   For Needle Providers: Does your profession involve administering needles to children?
   - ❑ Yes
   - ❑ No

5. Are you registered with your professional organization to work clinically in your province, state, or region?
   - ❑ Yes
   - ❑ No

6. Do you have access to a device and internet to view an e-resource?
   - ❑ Yes
   - ❑ No

7. Please select your current profession:
   - ❑ Child Life Specialist
   - ❑ Psychologist
   - ❑ Therapist
   - ❑ Social Worker
   - ❑ Counsellor
   - ❑ Nurse
   - ❑ Pharmacist
   - ❑ Physician
   - ❑ Other (describe): _______________

Caregiver-Child Dyad Screening Questionnaire

Basic inclusion criteria:

1. Are you currently living in Canada or the United States?
   - ❑ Yes
   - ❑ No

2. Do you have a child between the ages of 5 and 8 years old (birth years 2014 – 2017)?
   - ❑ Yes
   - ❑ No

3. Are you able to speak, write, and read English well enough to review an e-resource and participate in a discussion in English?
   - ❑ Yes
   - ❑ No

4. Is your child able to speak and understand English well enough to look at a picture book and participate in a simple discussion in English?
   - ❑ Yes
   - ❑ No

5. Are you one of the child's primary caregivers and would you be available to participate in an interview?
   - ❑ Yes
   - ❑ No

6. Do you have access to the internet and a device for viewing the e-resource?
   - ❑ Yes
   - ❑ No

Needle Fear:

7. On a scale from 0 to 10, where 0 is not at all afraid, 5 is moderately afraid and 10 is extremely afraid, how afraid do you think your child is of needles? ____

0	1	2	3	4	5	6	7	8	9	10
Not afraid at all										Extremely afraid

8. Does your child cry, scream, run away, have difficulty keeping still, or keep moving their arm away when they have to get a needle?

- ❑ Yes
- ❑ No

9. When your child is about to receive a needle, have you or a healthcare professional ever had to stop or delay the procedure because your child was so upset?
   - ❑ Yes
   - ❑ No

10. Do you ever delay or avoid needles for your child because of their fear?
    - ❑ Yes
    - ❑ No

Supplementary Material S2

Healthcare Professional Demographic Survey

1. Please indicate your current age______ (years)

2. Please indicate your gender____

3. Please indicate your ethnicity (check all that apply):

- ☐ First Nations/Metis/Inuit
- ☐ White/European
- ☐ Black/African/Caribbean
- ☐ Southeast Asian (e.g., Chinese, Japanese, Korean, Vietnamese, Cambodian, Filipino, etc.)
- ☐ Arab (Saudi, Palestinian, Iraqi, etc.)
- ☐ South Asian (East Indian, Sri Lankan, etc.)
- ☐ Latin American (Costa Rican, Guatemalan, Brazilian, Columbian, etc.)
- ☐ West Asian (Iranian, Afghani, etc.)
- ☐ Other (please specify):____
- ☐ Prefer not to say

Caregiver-Child Dyad Demographic Survey

1. What is your relationship to the child?
   - ☐ Mother
   - ☐ Father
   - ☐ Stepmother
   - ☐ Stepfather
   - ☐ Other____________________

2. Please indicate your current age______ (years) and gender____

3. Please indicate your ethnicity (check all that apply):

- ☐ First Nations/Metis/Inuit
- ☐ White/European
- ☐ Black/African/Caribbean
- ☐ Southeast Asian (e.g., Chinese, Japanese, Korean, Vietnamese, Cambodian, Filipino, etc.)
- ☐ Arab (Saudi, Palestinian, Iraqi, etc.)
- ☐ South Asian (East Indian, Sri Lankan, etc.)
- ☐ Latin American (Costa Rican, Guatemalan, Brazilian, Columbian, etc.)
- ☐ West Asian (Iranian, Afghani, etc.)
- ☐ Other (please specify):____
- ☐ Prefer not to say

Please answer the following about the child who is participating in the study.

4. Child's Age: ____ (years)

5. Child’s Gender: _______

6. Child's Date of Birth: ______ (month) ______ (year)

7. Please indicate your child’s ethnicity (check all that apply

- ☐ White/European
- ☐ First Nations/Metis/Inuit
- ☐ Black/African/Caribbean
- ☐ Southeast Asian (e.g., Chinese, Japanese, Korean, Vietnamese, Cambodian, Filipino, etc.)
- ☐ Arab (Saudi Arabian, Palestinian, Iraqi, etc.)
- ☐ South Asian (East Indian, Sri Lankan, etc.)
- ☐ Latin American (Costa Rican, Guatemalan, Brazilian, Columbian, etc.)
- ☐ West Asian (Iranian, Afghani, etc.)
- ☐ Prefer not to say
- ☐ Other (please specify):____

Supplementary Material S3

Table S1
Child-Caregiver Dyad Interview Guide

**Core Questions:**	**Potential Question-Specific Probes:**
**E-RESOURCE QUESTIONS (for caregivers alone)**	
1. So if you recall, we shared a first draft of a children's picture book with you as well as a guide for caregivers about needle fear.<br><br>What information **did you and your child learn** from the: a) children's picture book and b) caregiver-directed materials?	*- Please comment on your child's understanding of the picture book content. Was the language appropriate?*<br>*- Was anything surprising?*<br>*- Was this information new or something you had heard before?*<br>*- What resonated with your child?*
2. What were the **most helpful topic(s),** information, or activities that were covered in the: a) children's picture book and b) caregiver-directed materials?	*- Was there sufficient information covered on the topic?*<br>*- How was it helpful?*<br>*- Was there any information or content that you felt you would want to try for your child's fear?*
3. What were the **least helpful topic(**s) or activities that were covered in the: a) children's picture book and b) caregiver-directed materials?	*- In what way was it unhelpful? (e.g., confusing, unclear, needed more information or detail, language was not accessible)*<br>*- How could we make it better?*<br>*- If we were trying to shorter or take out content, what would you suggest?*<br>*- Is there anything else you just didn't like about the book or caregiver guide?*
4. What do you see as **the greatest challenges or barriers to using the e-resource?** (e.g., website not easy to use, caregivers not having enough time, not knowing that they should access something like this, not being child friendly enough?)	*- How can we address this barrier?*<br>*- What barriers came up for your child?*
5. Tell us how would you modify/change or what you would add to this tool?	*- What age range do you the think picture book is suited for?*<br>*- What would you change about the images to make them more child-friendly?*<br>*-What would you change about the book text?*<br>*- How could it be more engaging for your child?*<br>*- What would you take away from it?*<br>*- Is there any other information you would have liked to know?*<br>*- Visually how could the picture book be different?*<br>*- Visually what would you change about the e-resource*
**PICTURE BOOK QUESTIONS (for children and caregivers together)**	
For the following questions, the child will be asked first. Following the child answering each question, the caregiver will be provided with one of the following prompts:<br>*"Do you have anything to add?" or "Do you agree with that?"*	

Hey [Child's name]. Do you remember that book about needles that your mum/dad/caregiver read with you? Can I ask you a few questions about it? You can have it open if that will be helpful. If you think about that book…	
1. What did you learn about needles?	*- What did you learn about other children who are afraid of needles?*
2. What did you like most about the book?	*- Why was this your favourite part? Why?*<br>*- What was your favourite part? Anything cool about it?*
3. What did you NOT like about the book?	*- Why didn't you like it?*<br>*- Was any part of the book scary or hard to talk about?*<br>*- Was any part of the book scary or hard to see?*
4. The next time you go to get a needle, what are some things you can try to feel better?	*- Are there any things you would bring with you to get a needle?*<br>*- What would you want your big person or caregiver to be doing?*
5. If you could add or change anything about the book, what would it be?	*- Why would you change this?*<br>*- How can we make the book better for you?*<br>*- What would you change about the way it looks?*

Table S2

Healthcare Professional Interview Guide

**Core Questions:**	**Potential Question-Specific Probes:**
**CONTENT-SPECIFIC QUESTIONS**	
1. Tell us about the **most helpful** topic(s), information, or activities that were covered in the: a) children's picture book and b) caregiver-directed materials?	*- Was there sufficient information covered on the topic?*<br>*- What was helpful about it?*<br>*- do you have any positive or negative feedback on the way the materials are presented visually?*<br>*- What would you want to change?*
2. Tell us about the **least helpful** topic(s), information, or activities that were covered in the: a) children's picture book and b) caregiver-directed materials?	*- In what way was it unhelpful? (e.g., confusing, unclear, inaccurate, misleading, needed more information or detail, language was not accessible).*
3. What do you see as the greatest **challenges or barriers** to having caregivers of 5 to 8-year-old children with needle fear use the e-resource?	*- How can we address this barrier?*<br>*- Do you have any concerns about anyone's ability to use this e-resource?*<br>*- Developmentally where do you think the picture book is at right now?*<br>*If not, why and what changes would you recommend?*<br>*- How can we make it more child friendly?*
4. Tell us how would you **modify/change** or what you would add to the e-resource?	*- What would you add to it?*<br>*- What would you take away from it?*<br>*- Anything you would change visually?*<br>*- Is there any information you disagree with?*<br>*- Any information that could be misunderstood?*<br>*- Visually are there any changed you would make.*
5. How would you use this e-resource (if at all)?	*- (for child life specialists and mental health professionals) Would you use this resource when working with children with needle fear?*<br>*- Would you recommend this e-resource to other healthcare professionals?*

	-Would you recommend this e-resource for your patients/clients? If so, who would you recommend this to and why?
6. [For needle providers only] Can you speak to the e-resource's potential in **reducing children's PAIN** during vaccinations and venipunctures?	*- Was there sufficient information covered on pain management strategies?*<br>*- Are the pain management strategies depicted consistent with strategies you use when administering needles to children in North American health care settings?*<br>*- Why do you believe the e-resource may have potential to reduce pain?*<br>*- Why do you believe the e-resource may* ***not*** *have the potential to reduce pain?*
7. Please comment on the e-resource's potential in **reducing children's fear** during vaccinations and venipunctures?	*- Was there sufficient information covered managing fear and distress?*<br>*- Are the coping strategies depicted consistent with strategies you use when a needle is being administered?*<br>*- What might be missing in terms of helping kids with their fear?*

Supplementary Material S4

Healthcare Professional Feedback Survey

Please answer these questions to help us improve our e-resource. There are no right or wrong answers, we just want your opinion.

For questions 1 – 8, please share your feedback on the ***children's picture book*** *portion of the e-resource only.*

	**Strongly Disagree**				**Strongly Agree**
1. The information was accurate.	1	2	3	4	5
2. The picture book has a good amount of detail.	1	2	3	4	5
3. The information was presented in an organized way.	1	2	3	4	5
4. The information was presented in an accessible way (e.g., information was clear, understandable).	1	2	3	4	5
5. I would recommend this resource to other healthcare professionals	1	2	3	4	5
6. I would recommend this resource to my patients and clients	1	2	3	4	5

7. The amount of information was: ☐ Too much ☐ Right amount ☐ Not enough

8. Please indicate your overall level of satisfaction with the picture book: (select one)

1	2	3	4	5
Very dissatisfied	Moderately dissatisfied	Neutral	Moderately satisfied	Very satisfied

For questions 9 – 16, please share your feedback on the ***parent resource*** *portion of the e-resource only.*

	**Strongly Disagree**				**Strongly Agree**
9. The information was accurate.	1	2	3	4	5
10. The parent resource has a good amount of detail.	1	2	3	4	5

11. The information was presented in an organized way.	1	2	3	4	5
12. The information was presented in an accessible way (e.g., information was clear, understandable).	1	2	3	4	5
13. I would recommend this resource to other healthcare professionals	1	2	3	4	5
14. I would recommend this resource to my patients and clients	1	2	3	4	5

15. The amount of information was: ☐ Too much ☐ Right amount ☐ Not enough

16. Please indicate your overall level of satisfaction with the parent resource: (select one)

1	2	3	4	5
Very dissatisfied	Moderately dissatisfied	Neutral	Moderately satisfied	Very satisfied

For the remaining items, please share your feedback on the e-resource ***<u>as a whole</u>*** *(children's picture book + parent resource).*

17. Please estimate the amount of time you spent reviewing the e-resource (in minutes):

____

18. How likely are you to recommend this resource to parents of children with moderate to high levels of needle fear?

1	2	3	4	5
Very unlikely	Unlikely	Unsure	Likely	Very likely

   a. If you indicated "very unlikely", "unlikely", or "unsure" please explain why: __________

19. How likely do you think your clients/patients would be to follow the recommendations in the e-resource?

1	2	3	4	5
Very unlikely	Unlikely	Unsure	Likely	Very likely

If you indicated “very unlikely”, “unlikely”, or “unsure” please explain why: __________

20. What did you like **most** about the e-resource?

____

____

21. What did you like **least** about the e-resource?

____

____

22. How could this e-resource be improved? (Other topics? Format?)

____

____

23. Was there anything that you think was inaccurate in the e-resource?

____

____

24. Are there any other comments/feedback you would like to share that we did not ask about?

____

____

Thank you for your feedback!

Caregiver-Child Dyad Feedback Survey

Please answer these questions to help us improve our e-resource. There are no right or wrong answers, we just want your opinion.

For questions 1 – 7, please share your feedback on the ***children's picture book*** *portion of the e-resource only.*

	Strongly Disagree				Strongly Agree
25. The information was applicable to my child.	1	2	3	4	5
26. The picture book had a good amount of detail.	1	2	3	4	5
27. The information was presented in an organized way.	1	2	3	4	5
28. The information was presented in an accessible way (e.g., information was clear, understandable).	1	2	3	4	5
29. I would recommend this resource to other parents of children who are afraid of needles	1	2	3	4	5

30. The amount of information was: ☐ Too much ☐ Right amount ☐ Not enough

31. Please indicate your overall level of satisfaction with the picture book: (select one)

1	2	3	4	5
Very dissatisfied	Moderately dissatisfied	Neutral	Moderately satisfied	Very satisfied

For questions 8 – 14, please share your feedback on the ***parent resource*** *portion of the e-resource only.*

	Strongly Disagree				Strongly Agree
32. The information was applicable.	1	2	3	4	5
33. The parent resource has a good amount of detail.	1	2	3	4	5
34. The information was presented in an organized way.	1	2	3	4	5
35. The information was presented in an accessible way (e.g., information was clear, understandable).	1	2	3	4	5

36. I would recommend this resource to other parents of children with needle fear 1 2 3 4 5

37. The amount of information was: ☐ Too much ☐ Right amount ☐ Not enough

38. Please indicate your overall level of satisfaction with the parent resource: (select one)

1	2	3	4	5
Very dissatisfied	Moderately dissatisfied	Neutral	Moderately satisfied	Very satisfied

For the remaining items, please share your feedback on the e-resource ***<u>as a whole</u>*** *(children's picture book + parent resource).*

39. Please estimate the amount of time you spent reviewing the e-resource (in minutes): ________________

40. How likely are you to recommend this resource to other parents of children with moderate to high levels of needle fear?

1	2	3	4	5
Very unlikely	Unlikely	Unsure	Likely	Very likely

   a. If you indicated "very unlikely", "unlikely", or "unsure" please explain why: __________

41. How likely do you think other parents would be to follow the recommendations in the e-resource?

1	2	3	4	5
Very unlikely	Unlikely	Unsure	Likely	Very likely

If you indicated "very unlikely", "unlikely", or "unsure" please explain why: __________

42. What did you like **<u>most</u>** about the e-resource?

____

____

43. What did you like **least** about the e-resource?

____

____

44. How could this e-resource be improved? (Other topics? Format?)

____

____

45. Was there anything that you think was inaccurate in the e-resource?

____

____

46. Are there any other comments/feedback you would like to share that we did not ask about?

____

____

Thank you for your feedback!
